# Collapse of Cytolytic Potential in SIV-Specific CD8+ T Cells Following Acute SIV Infection in Rhesus Macaques

**DOI:** 10.1371/journal.ppat.1006135

**Published:** 2016-12-30

**Authors:** Emily R. Roberts, Diane G. Carnathan, Hui Li, George M. Shaw, Guido Silvestri, Michael R. Betts

**Affiliations:** 1 Department of Microbiology, University of Pennsylvania, Philadelphia, Pennsylvania, United States of America; 2 Biomedical Graduate Studies in Immunology, University of Pennsylvania School of Medicine, Philadelphia, Pennsylvania, United States of America; 3 Emory Vaccine Center, Yerkes National Primate Research Center, Emory University, Atlanta, Georgia, United States of America; University of Wisconsin, UNITED STATES

## Abstract

Poor maintenance of cytotoxic factor expression among HIV-specific CD8+ T cells, in part caused by dysregulated expression of the transcription factor T-bet, is associated with HIV disease progression. However, the precise evolution and context in which CD8+ T cell cytotoxic functions become dysregulated in HIV infection remain unclear. Using the rhesus macaque (RM) SIV infection model, we evaluated the kinetics of SIV-specific CD8+ T cell cytolytic factor expression in peripheral blood, lymph node, spleen, and gut mucosa from early acute infection through chronic infection. We identified rapid acquisition of perforin and granzyme B expression in SIV-specific CD8+ T cells in blood, secondary lymphoid tissues and gut mucosa that collapsed rapidly during the transition to chronic infection. The evolution of this expression profile was linked to low expression of T-bet and occurred independent of epitope specificity, viral escape patterns and tissue origin. Importantly, during acute infection SIV-specific CD8+ T cells that maintained T-bet expression retained the ability to express granzyme B after stimulation, but this relationship was lost in chronic infection. Together, these data demonstrate the loss of cytolytic machinery in SIV-specific CD8+ T cells in blood and at tissue sites of viral reservoir and active replication during the transition from acute to chronic infection. This phenomenon occurs despite persistent high levels of viremia suggesting that an inability to maintain properly regulated cytotoxic T cell responses in all tissue sites enables HIV/SIV to avoid immune clearance, establish persistent viral reservoirs in lymphoid tissues and gut mucosa, and lead ultimately to immunopathogenesis and death.

## Author Summary

The underlying reasons why HIV avoids immune-mediated clearance and establishes chronic infection remain undefined. Poor cytotoxic CD8+ T cell (CTL) killing functions and localization of CD8+ T cells to critical anatomical sites associated with virus replication and reservoir have been described during chronic HIV infection. However, it is not known how CTL functions develop during primary HIV infection. Using the SIV infection model in rhesus macaques, we characterized the expression of cytolytic factors in virus-specific CTL in blood and at critical anatomical sites, including lymph nodes, spleen and gut mucosa, throughout acute infection. We found high expression of cytolytic factors in CD8+ T cells during acute infection in blood, lymph nodes, spleen and gut mucosa that was short-lived despite persistence of virus. We also found the highest levels of cytolytic factors in CTL in the blood as compared to critical anatomical sites in the earliest detectable CTL responses that was associated with expression of the regulatory factor T-bet. Our data describe short-lived CTL cytolytic factor expression declining as early as the acute phase of infection. Our study contributes to understanding HIV/SIV persistence and how virus avoids CTL-mediated clearance in lymphoid tissue and gut mucosa.

## Introduction

While it is clear that CD8+ T cells are a critical component of the host immune response to human immunodeficiency virus (HIV) and simian immunodeficiency virus (SIV) control, these cells only rarely fully control virus replication (i.e., elite controllers) [1–3]. The reasons why CD8+ T cells do not control HIV/SIV replication in the majority of infected individuals remain unclear. Seminal work directly identifying the importance of CD8+ T cells in partial containment of viremia was performed in SIV infected rhesus macaques (RM), whereby depletion of CD8+ T cells consistently resulted in significant increases in viremia and accelerated disease progression [4, 5]. In addition, *in vivo* viral evolution studies revealed the emergence of escape mutants for the most common immunodominant CD8+ T cell viral epitopes of SIV, thus demonstrating a significant level of immune pressure exerted on the virus by CD8+ T cells [6, 7]. More recently, CD8+ T cell depletion studies in elite controller SIV infected RM indicated the requirement of CD8+ T cell persistence in lymph nodes (LN) and spleen to maintain control of plasma viremia and productively infected CD4+ T cells in these tissues [8, 9].

Several effector mechanisms have been proposed to explain how CD8+ T cells mediate viral control, including cytolytic and non-cytolytic processes [10–12]. Granule-mediated cytolysis is a rapid and powerful effector response used by cytotoxic CD8+ T lymphocytes (CTL) to clear target cells in an antigen-specific manner [13, 14]. This process includes two necessary factors, a pore forming component, perforin, which enables entry of a protease, most often granzyme B, to induce programmed cell death through both caspase-dependent and caspase-independent induction of apoptosis [13]. The induction and maintenance of granule-mediated cytolysis, including the expression of perforin and granzyme B has been shown to be in part mediated by the transcription factor T-bet, where in its absence CD8+ T cell effector functions are significantly reduced [15–21]. Previous studies have shown that the maintenance of CD8+ T cell killing capacity is a key factor in control of HIV infection, as measured by granzyme B and perforin expression and expression levels of T-bet [15, 22–25]. These studies highlight the relationship of low levels of viremia in HIV elite controllers with the presence of robust T-bet controlled cytotoxic T lymphocyte (CTL) killing potential by HIV-specific CD8+ T cells that is present through chronic phase of infection. This is further supported by a recent study from our group describing the evolution of HIV-specific CD8+ CTL during acute infection and identified the loss of T-bet and concurrent declining perforin expression as key markers of the transition from acute to the chronic phase of HIV infection [18]. Virus-specific CD8+ T cell non-cytolytic mechanisms mediated by β-chemokines, α-defensins, and the unclassified suppressive factor, CAF, have been evaluated albeit less extensively than cytolytic mechanisms during HIV/SIV infection [26–29]. Both Mip-1α and α-defensin productions by CD8+ T cells are positively correlated with control of HIV viremia in elite controller subjects, although their role is likely more involved in blocking receptor binding and subsequent infection [29–31]. Nevertheless, these factors are thought to contribute to control of viremia without elimination of infected cells [11, 12].

Secondary lymphoid tissues (SLT) and gut mucosa are key sites of HIV/SIV replication and reservoirs during the acute and chronic phases of infection [32–35]. In particular, follicular CD4+ T helper cells harbor high levels of integrated provirus and are major contributors to both ongoing viral replication and virus rebound observed following interruption of antiretroviral therapy [32, 33]. Studies detecting virus-specific CD8+ T cells from SLT and gut mucosa have been focused primarily on the chronic phase of infection with few studies investigating the early acute phase [34, 36–39]. As such, it remains unclear to what degree virus-specific CD8+ T cells in these compartments possess cytolytic function when the reservoir is first seeded and subsequently maintained. These studies underscore the need to evaluate virus-specific CD8+ CTL potential in SLT and gut mucosa in the earliest detectable responses in acute infection, as well as those present during chronic infection.

In the current study we sought to address these issues through sequential necropsy conducted during the acute phase of infection in SIV infected macaques to longitudinally dissect the evolution of cytolytic CD8+ T cell function in blood and at relevant sites of viral replication, including SLT and gut. To this end, we infected Mamu A*01-positive RM with SIV_mac251_ and longitudinally monitored the CD8+ T cell responses to two immunodominant epitopes, Tat-TL8 and Gag-CM9, for their acquisition and modulation of cytolytic factors granzyme B and perforin in blood, spleen, LN and gut mucosa through the earliest stages of acute infection and up to chronic infection. We found that the initial high expression of cytolytic factors was remarkably short-lived, and collapsed rapidly during late acute infection in nearly all animals. This phenomenon resulted in low levels of cytolytic SIV-specific CD8+ T cell responses during the chronic phase of infection despite high plasma viremia. Notably, SIV-specific CD8+ T cells in LN consistently expressed lower levels of perforin, and granzyme B as well as T-bet compared to their peripheral blood counterparts. These findings demonstrate the rapid collapse of a dominant cytolytic profile among SIV-specific CD8+ T cells during acute SIV infection in all tissue compartments, most importantly in SLT where active seeding of SIV reservoir and viral replication is initiated and persists during chronic infection.

## Materials and Methods

### Ethical statement for use of non-human primates

Procedures carried out in this study were performed following an Institutional Animal Care and Use Committee (IACUC) of Emory University approved protocol, number #YER-2002541-121716GA in accordance with the Animal Welfare Act and other federal statutes and regulations relating to animals. All animals were housed at the Yerkes National Primate Research Center (YNPRC) at Emory University (Atlanta, GA) following the guidelines established by the National Institutes of Health (NIH), under the supervision of the Association for the Assessment and Accreditation of Laboratory Animal Care (AAALAC)-accredited Division of Animal Resources. Housing and care for all animals complied with and met the standards established and written in the Animal Welfare Act, Animal Welfare Regulations, and The Guide for the Care and Use of Laboratory Animals (8thEdition). All tissue sample collections were performed under anesthetization using Ketamine (10 mg/kg IM) or Telazol (5 mg/kg IM) to minimize suffering. No pre-scheduled sacrifice or death of animals was occured in this study. Reduction of stress was provided through enrichment activities such foraging for grains, Kong toys, as well as other enrichment strategies to animals in need as determined by the YNPRC veterinary and/or care staff. Additionally, animals were housed in paneled cages so as to provide some social interaction and contact with one another including grooming and other social activities, when feasible.

### Animals

**Fig 1:**
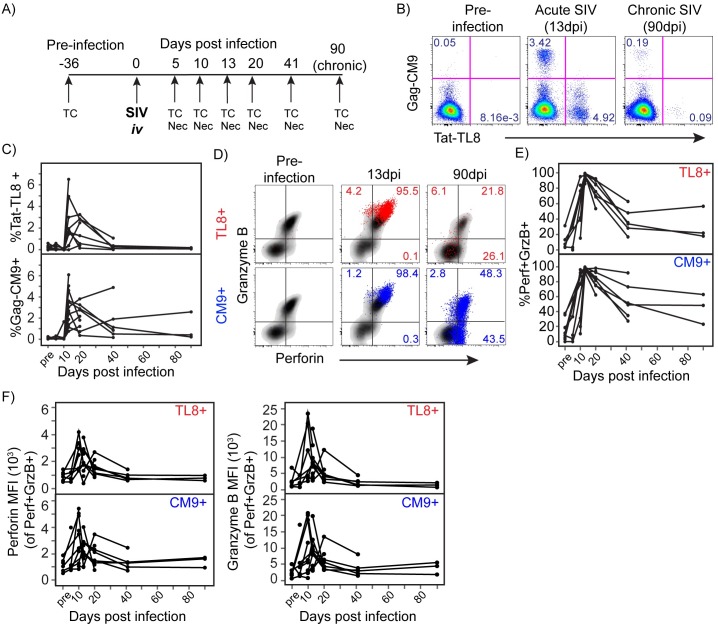
Evolution of CTL in peripheral blood during acute SIV infection. A) Experimental design including SIV infection, tissue and blood sample collection (TC), and necropsy (Nec) time points. B) Flow cytometry plots of Tat-TL8 and Gag-CM9 Mamu A*01 tetramer staining in CD8+ T cells at pre-infection, acute, and chronic infection from a representative animal. Frequencies of single positive tetramer+ events are shown. C) The frequency of TL8 and CM9 tetramer+ SIV-specific CD8+ T cells in peripheral blood CD8+ T cells throughout infection. D) Flow cytometry plots of perforin and granzyme B expression in TL8 and CM9 tetramer+ SIV-specific CD8+ T cells (TL8 tetramer+ in red, and CM9 tetramer+ in blue) overlayed on total CD8+ T cells (black) at pre-infection, 13dpi, and 90dpi from representative animals. Frequencies of gated populations are shown of TL8 or CM9 tetramer+ cells. E) The frequency of perforin and granzyme B (Perf+GrzB+) co-expression among TL8 or CM9 tetramer+ SIV-specific CD8+ T cells throughout infection. F) The median fluorescence intensity (MFI) of perforin and granzyme B in Perf+GrzB+, tetramer+ SIV-specific CD8+ T cells throughout infection. Lines connecting data points represent longitudinally collected data from individual animals.

Eighteen healthy, SIV-uninfected, Mamu-A*01-positive (MHC-class I) Indian rhesus were used in this study. RM selected for this study were 32–38 months old (males (n = 13) and females (n = 5)), and Mamu B*08 and Mamu B*17-negative to exclude animals with predispositions for control of viremia. Animals selected for the study were quarantined for 30–45 days in accordance with the YNPRC IACUC prior to the start of the study. Animals underwent staggered necropsy post SIV infection during acute infection at 5, 10, 13, 20, or 41 days post-infection (dpi), or during chronic infection at 90dpi. Three animals were sacrificed at each indicated time point post-infection. (Fig 1a and S1a Fig).

### Infection and monitoring viral load

All animals were infected with _500_TCID_50_ SIV_mac251_ intravenous (*iv*) (10.8ng/mL p27) (Nancy Miller, NIAID, NIH). Whole blood was collected by venous puncture into EDTA blood collection tubes (BD, Franklin Lakes, NJ) for evaluating SIV viral loads. Plasma viremia was monitored longitudinally in all animals using quantitative real time PCR, with sensitivity of 60 copies/mL by the Emory University CFAR Virology Core facilities as previously described [40].

### Viral sequencing and analysis

Blood plasma from two animals was evaluated between 10dpi and 90dpi for Tat-TL8 and Gag-CM9 epitope sequence monitoring. Virus inoculum stock was evaluated to determine the frequencies of each epitope present at the time of infection. Between 40,000–80,000 vRNA copies were extracted from blood plasma samples and viral inoculum stock using the Qiagen BioRobot EZ1 Workstation with EZ1 Virus Mini Kit v2.0 (Qiagen, Valencia, CA). vRNA was subject to reverse transcription using MuLV (SuperScript III) reverse transcriptase using the following primers; Tat-RT3: 5’–TGGGGATAATTTTACACAAGGC-3’ [41] and SMgag.R1: 5’-CTACTGGTCTTCTCCAAAGAGAGAATTGA-3’ for analysis of TL8 and CM9 epitopes, respectively. Resampling of PCR products was avoided by obtained PCR end point dilutions of each cDNA sample as previously described [42]. Equal quantities of cDNA from each sample were PCR amplified to achieve the desirable sampling depth. Multiple bulk PCR reactions were performed and subsequently pooled to reach sufficient cDNA volumes when necessary. cDNA samples were analyzed using MiSeq analysis and amplification fragment lengths of roughly 2,035bp and 1,560bp were generated for TL8 and CM9 epitope coding regions, respectively. PCR primers used for TL8 region amplification are as follows; 1^st^ round, Fwd: smE660/mac251-int-F1 5’-ATGAATTTTAAAAGAAGGGGAGGAA-3’, Rev: Tat-R2 5’-CCCAAGTATCCCTATTCTTGGTTGCAC-3’ [41] and 2^nd^ round, Fwd: smE660/mac251-int-F2: 5’-AAGAAGGGGAGGAATAGGGGATATG-3’, Rev: Tat-R3 5’-GAGCAAGATGGCGATAAGCAG-3’ [41]. PCR primers used for CM9 region amplification are as follows; 1^st^ round, Fwd: SIV.R.F1 5’-TCGCTCTGCGGAGAGGCTG-3’, and Rev: SMgag.R1 5’-CTACTGGTCTTCTCCAAAGAGAGAATTGA-3’, and 2^nd^ round, Fwd: SIV.R.F2 5’- TGCGGAGAGGCTGGCAGATTGA-3’, Rev: SM.gag.R2 5’-TCCAAAGAGAGAATTGAGGTGCAGCAA-3’. Raw sequence reads were translated into amino acid sequences and aligned to the SIVmac251 reference sequence. Variations in Tat-TL8 and Gag-CM9 epitopes were determined as compared to canonical Tat-TL8 (TTPESANL) and Gag-CM9 (CTPYDINQM) reference amino acid sequences. The frequency of each detected epitope variant was determined using Geneious R9 software (Geneious, Auckland, New Zealand). Changes in the distribution of canonical Tat-TL8 and Gag-CM9 sequences between virus inoculum stock and chronic phase of infection, at 90dpi, were monitored (S2a and S2b Fig).

### Tissue collection and lymphocyte cryopreservation

All samples were collected at YNPRC and shipped overnight to the University of Pennsylvania for processing the following day, with the exception of peripheral blood collected for complete blood count and blood plasma viral load analysis. Tissues were collected from each animal at the indicated time points pre- and post-infection (Fig 1a and S1a Fig). Peripheral blood, axillary and inguinal superficial lymph nodes (sLN), and rectal biopsies (RB) were collected longitudinally from each animal, while spleen and mesenteric lymph nodes (mLN) were collected at necropsy only. Peripheral blood was collected by venous puncture into EDTA or sodium heparin blood collection tubes (BD, Franklin Lakes, NJ). Blood collection volumes were taken in the IACUC approved sample volumes at the indicated time points pre- and post SIV infection. Superficial LN samples were collected under general anesthesia by surgery at the indicated time points pre- and post SIV infection. Samples were stored in sterile R-10 media (Rosewell Park media (RPMI) (Mediatech Inc, Manassas, VA), supplemented with 10% fetal bovine serum (FBS) (Gemini Bio-Products, West Sacramento, CA), Penicillin/Streptomycin, L-Glutamine, and kept at 2–8°C until processing. RBs were collected under general anesthesia using a sigmoidoscope at the indicated time points pre- and post SIV infection. Samples were stored in sterile R-10 media and kept at 2–8°C until processing. Mesenteric LNs, remaining sLN, and spleen were collected at necropsy only. Samples were stored in sterile R-10 media and kept at 2–8°C until processing.

### Processing tissues

All tissue processing was done at the University of Pennsylvania in accordance with University Environmental and Health Safety Guidelines under sterile conditions. Rectal biopsies were processed to extract intraepithelial and lamina propria residing lymphocytes by the University of Pennsylvania Human Immunology Core facility. The extraction process included serial washes of biopsies with R-10 media plus collagenase XI followed by percoll 65/30 gradient for separation of lymphocytes from contaminating epithelial cells. Peripheral blood mononuclear cells were isolated from venous whole blood collected using standard Ficoll-Paque PLUS (GE Healthcare, Pittsburgh, PA) diluted to 90% in 1X Dulbecco’s Phosphate Buffered Saline (Mediatech Inc, Manassas, VA), and centrifuged at 350g for 20 minutes. Spleen and LN were processed by mechanical disruption in 2% FBS (Gemini Bio-Products, West Sacramento, CA) in Dulbecco’s Phosphate Buffered Saline with 10U/mL of DNAse I (Roche Life Sciences, Indianapolis, IN) for extraction of lymphocytes. Splenocytes were subsequently treated with sterile ACK red cell lysis buffer (NH_4_Cl 0.15M, KHCO_3_ 10mM, EDTA 0.1mM pH 7.2–7.4 in distilled deionized water). All extracted lymphocytes with the exception of those extracted from RBs were immediately cryopreserved in 10% DMSO (Fisher Scientific, Fair Lawn, NJ) in FBS (Gemini Bio-Products, West Sacramento, CA) and stored at -150°C. All RB derived lymphocytes were evaluated fresh.

### Lymphocyte stimulation

Cryopreserved lymphocytes from peripheral blood, mLN, sLN, and spleen were thawed and rested overnight in sterile R-10 media plus 10U/mL DNAse I (Roche Life Sciences, Indianapolis, IN) at 37°C, 5% CO_2_ and 95% humidity incubation conditions. Lymphocytes from blood, sLN, mLN and spleen were stimulated as described in [43] at 1.0mL final volume in sterile R-10 media. Peptide concentrations for stimulation conditions were 2μg/mL Tat-TL8 peptide (TTPESANL) or Gag-CM9 (CTPYDINQM) (New England Peptide, Gardner, MA). Co-stimulation was added with peptides; 1μg/mL anti-CD49d (Clone 9F10, Biolegend, San Diego, CA) and CD28-ECD (Clone CD28.2, Beckman Coulter, Brea, CA) at the start of stimulation. Positive control samples were stimulated using Staphylococcal Enterotoxin B (List Biological Laboratories, Campbell, CA) at 1μg/mL. CD107a-BV421 (Biolegend, San Diego, CA) was added at the start of stimulation. Brefeldin A (1μg /mL) (Sigma Aldrich, Saint Louis, MO) and monensin (0.66μL/mL) (BD Bioscience, San Jose, CA) were added one hour after initiation of stimulation. Cells were incubated under stimulation conditions for a total of 9 hours.

### MHC class I tetramers

Tat-TL8 (TTPESANL) Mamu A*01-APC conjugated tetramers and Gag-CM9 (CTPYDINQM) Mamu A*01 monomers were supplied by the NIH Tetramer Core Facility at Emory University, Atlanta, GA. Gag-CM9 Mamu A*01-BV421 tetramers were prepared in our laboratory using Gag-CM9 momomers and streptavidin-BV421 (Biolegend, San Diego, CA) with protease inhibitor cocktail set I (Calbiochem, Billerica, MA).

### Fluorescence cytometry staining

Lymphocytes were stained as described in [43]. MHC class I, Mamu A*01 tetramer staining was done at 37°C at 5% CO_2_ and 95% humidity for 10 minutes, concurrent with CCR7 staining when applicable, prior to the addition of viability dye and remaining antibodies for immunophenotype staining.

### Antibodies

The following antibodies were used for phenotyping and functional evaluation of lymphocytes in this study: CD107a-BV421 (Clone H4A3, Biolegend), CD4-PECy5.5 (Clone S3.5, Invitrogen), CD8-BV570 (Clone RPA-T8, Biolegend), CD14-BV650 (Clone M5E2, Biolegend), CD20-BV650 (Clone 2H7, Biolegend), CD16-BV650 (Clone 3G8, Biolegend), CD28-ECD (Clone CD28.2, Beckman Coulter), CD95-PECy5 (Clone Dx2, BD Biosciences), CD38-PE (Clone OKT10, NIH NHP Reagent Resource, University of Massachusetts, Boston, MA), CCR7- BV711 (Clone G043H7, Biolegend). CD3-APCCy7 (Clone SP34-2, BD Biosciences), granzyme B-AF700 (Clone GB11, BD Biosciences), T-bet-PECy7 (Clone 4B10, eBioscience), Ki67-FITC (Clone B56, BD Bioscience) or Ki67-BV786 (Clone B56, BD Bioscience), CD69-APC (Clone FN50, BD Bioscience) or CD69-BV605 (Clone FN50, Biolegend), Perforin-FITC (Clone pf344, MabTech), TNF-BV605 (Clone MAb11, Biolegend), IFNg-BV785 (Clone 4S.B3, Biolegend), Live Dead Fixable Aqua Dead Cell Stain (Molecular Probes).

### Data acquisition and analysis

All immunofluorescence stained lymphocytes were acquired and analyzed on a special order LSR II Flow cytometer using BD FACS Diva Software version 6.0.1 or 8.0.1 (Becton Dickinson, San Jose, CA). Flow cytometry data was analyzed using FlowJo version 9.8.3 (Treestar, Ashland, OR). Statistical analysis and graphs of flow cytometry data were performed using JMP Pro 12 (SAS, Cary, NC) or Prism v5.0 (GraphPad Software, La Jolla, Ca). All reported values for functional response after peptide stimulation were done with background (unstimulated) levels subtracted and with a minimum of 10 events for functional response or 25 events for tetramer responses. Analysis of paired samples, longitudinally or between matched tissues at a single time point was done by paired student t-test with alpha 0.05. Regression ANOVA was done on samples collected between 13dpi and 90dpi to monitor cross sectional changes over time. * indicates a p<0.05, ** indicates a p value <0.01, and *** indicates a p value <0.001.

## Results

### Induction and resolution of cytolytic SIV-specific CD8+ T cell responses during acute SIV infection

To define the acute kinetics of the CD8+ T cell response in SIV infection, we infected n = 18 RM with _500_TCID_50_ SIV_mac251_ *iv* and performed a timed tissue collection and necropsy protocol through chronic infection. We collected blood, spleen, mesenteric lymph nodes (mLN), superficial lymph nodes (LN), and rectal biopsies (RB) at the indicated time points pre- and post-infection (Fig 1a and S1a Fig). We infected all animals with a high dose of SIV_mac251_ virus in order to synchronize the infection and peak viremia. As shown in S1b Fig, the kinetics of viremia were uniform and reproducible in all animals. As expected, viremia in these animals peaked between 10 and 13dpi (Range: 1.5e^7^–1.06e^8^), and high viral loads persisted until necropsy. No animals showed signs of controlled viremia during the transition from acute to chronic phase of infection. As expected, during acute infection, absolute CD4+ T cells counts decreased rapidly and remained low in all animals (S1c Fig), and the percentage of CD4+ T cells in the gut mucosa declined precipitously within the first 5dpi, and remained very low in all animals (S1d Fig) [44].

To assess the induction of cytolytic SIV-specific CD8+ T cell responses, we first evaluated the responses to the immunodominant Mamu A*01 restricted epitopes Tat-TL8 and Gag-CM9 in peripheral blood. As shown in Fig 1b and 1c, CD8+ T cells specific for TL8 or CM9 epitopes peaked at 13dpi, followed by contraction into the chronic phase of infection as previously been described [6]. We next evaluated the expression of the cytolytic proteins perforin and granzyme B by SIV-specific CD8+ T cells. We found high co-expression of both molecules in Tat-TL8 and Gag-CM9 specific CD8+ T cells in blood that peaked at 13dpi, as shown in Fig 1d and 1e. This cytolytic profile was short-lived, however, with less than 50% on average of SIV-specific CD8+ T cells maintaining perforin and granzyme B co-expression beyond 21dpi. The collapse in co-expression of perforin and granzyme B occurred independent of epitope specificity as seen in both TL8 and CM9-specific responses (Fig 1e). Perforin and granzyme B expression on a per-cell basis peaked at 13dpi of the acute phase and similarly decreased as animals progressed into chronic infection (Fig 1f).

Previous studies documented the rapid and complete escape of the Tat-TL8 epitope (TTPESANL) during acute infection [6]. We therefore sought to confirm this finding as it relates to potential pressure mediated by cytolytic SIV-specific CD8+ T cells. Escape of the Tat-TL8 epitope, present in more than 50% of SIV_mac251_ inoculum virus (S2a Fig), occurred during the peak cytolytic TL8-specific response as seen in S2b and S2c Fig. However, the Gag-CM9 epitope did not escape despite similar CD8+ T cell mediate cytotoxic pressure, likely due to fitness constraints on this epitope as discussed previously (S2b and S2c Fig) [45]. Together these data show that the initial circulating CD8+ T cell responses to SIV infection have high expression of cytolytic molecules that is rapidly lost as infection transitions into the chronic phase.

### Rapid loss of cytolytic CD8+ T cells from secondary lymphoid tissues

**Fig 2:**
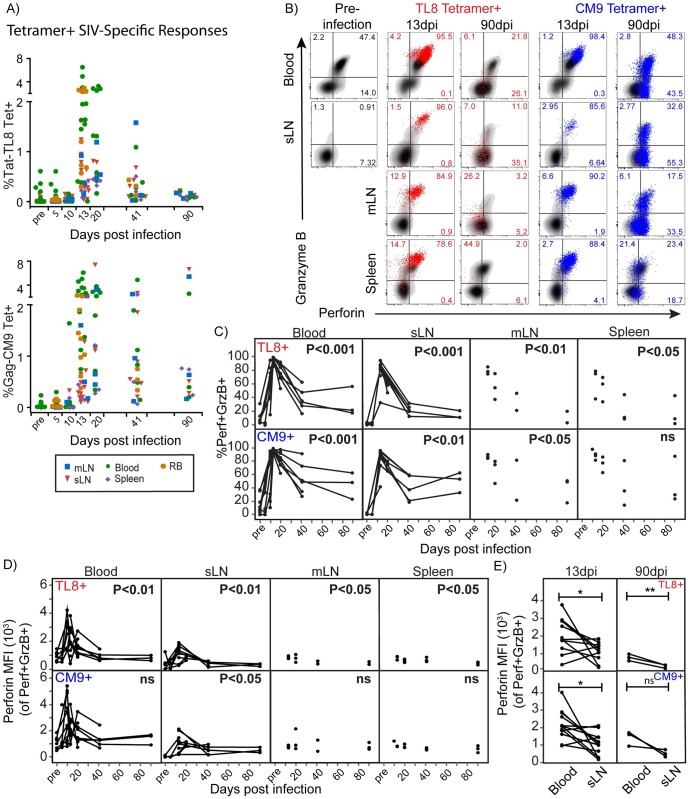
Evolution of CTL in blood, SLT and gut mucosa during acute SIV infection. A) The frequencies of TL8 and CM9 tetramer+ SIV-specific CD8+ T cells among CD8+ T cells in mLN, sLN, spleen, gut mucosa and blood at the indicated time points. B) Flow cytometry plots of perforin and granzyme B expression in TL8 and CM9 tetramer+ SIV-specific CD8+ T cells (TL8 tetramer+ in red, and CM9 tetramer+ in blue) overlayed on total CD8+ T cells (black) in blood, sLN, mLN and spleen at pre- infection, 13dpi, and 90dpi from representative animals. Frequencies of gated populations are shown of TL8 or CM9 tetramer+ cells. C) The frequencies of Perf+GrzB+ co-expression in TL8 and CM9 tetramer+ SIV-specific CD8+ T cells from blood, sLN, mLN and spleen at the indicated time points throughout infection; Linear regression ANOVA of samples collected between 13dpi and 90dpi. D) Kinetics of perforin median fluorescence intensity (MFI) in Perf+GrzB+ TL8 and CM9 tetramer+ SIV-specific CD8+ T cells at the indicated time points throughout infection; Linear regression ANOVA of samples collected between 13dpi and 90dpi. E) Perforin MFI in Perf+GrzB+ tetramer+ cells in sLN and blood at 13dpi or 90dpi; Paired, one-tailed Student’s t-test; 13dpi (n = 12 pairs) or 90dpi (n = 3 pairs). Lines connecting data points represent longitudinally collected data from sLN and blood of individual animals. * p≤0.05, ** p<0.01. No significant differences “ns”.

To assess the evolution of cytolytic SIV-specific CD8+ T cells at relevant sites of viral replication and reservoir, we next examined Tat-TL8 and Gag-CM9 specific CD8+ T cell responses in LN, spleen and gut mucosa tissue throughout acute infection. Tat-TL8 and Gag-CM9-specific CD8+ T cell responses were readily detectable in LN, spleen and gut mucosa as previously described (S3 Fig) [46, 47], peaking at 13dpi in all measured tissues, and underwent contraction in all tissues while SIV infection transitions into its chronic phase (Fig 2a).

Co-expression of perforin and granzyme B was detectable in SIV-specific CD8+ T cells in sLN, mLN, and spleen (Fig 2b) and peaked at 13dpi in all measured tissues (Fig 2c). SIV-specific CD8+ T cells in gut mucosa also showed very high granzyme B expression at peak viremia in nearly all animals (perforin not measured; S4a and S4b Fig). However, following peak responses, we observed a rapid loss of perforin and granzyme B co-expression within TL8 and CM9-specific CD8+ T cells in 8 out of 9 animals by 41dpi, shown in Fig 2c. We also observed declining granzyme B expression in SIV-specific CD8+ T cells in gut mucosa as animals transitioned to chronic infection (S4b Fig).

We next performed a quantitative analysis of the expression levels of perforin and granzyme B by median fluorescence intensity (MFI) in the subset of SIV-specific CD8+ T cells co-expressing perforin and granzyme B (Fig 2d and S5 Fig). Similar to blood (Fig 1f), perforin MFI peaked at 13dpi in SIV-specific CD8+ T cells in sLN but showed limited expression throughout infection in mLN and spleen (Fig 2d). Perforin expression was reduced in SIV-specific CD8+ T cells from sLN compared to blood at peak response and in TL8-specific CD8+ T cells during chronic infection (Fig 2e). Lower perforin expression levels were detected in CM9-specific CD8+ T cells in 2 out of 3 animals at chronic infection. Granzyme B within SIV-specific CD8+ T cells from LN and spleen largely mirrored the expression level of perforin in blood over time, peaking at 13dpi followed by a decline in expression levels during the transition to chronic infection (S5 Fig). Collectively, these data indicate short-lived cytolytic profiles in SIV-specific CD8+ T cell responses from the blood, SLT and gut mucosa during acute infection.

### Progressive loss of T-bet expression within SIV-specific CD8+ T cells in acute SIV infection

**Fig 3:**
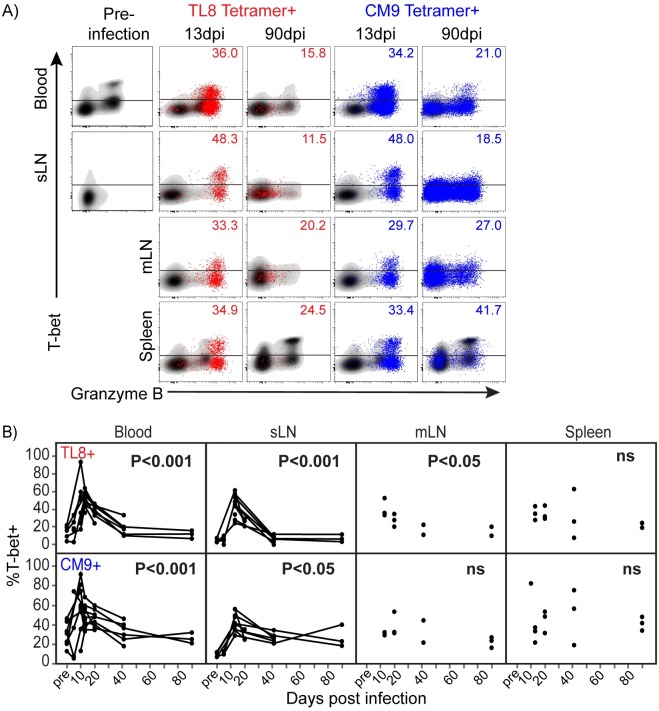
Kinetics of T-bet expression in SIV-specific CTL during acute SIV infection. A) Flow cytometry plots of T-bet expression in TL8 and CM9 tetramer+ SIV-specific CD8+ T cells (TL8 tetramer+ in red, and CM9 tetramer+ in blue) overlayed on total CD8+ T cells (black) in blood, sLN, mLN, and spleen at the indicated time points from representative animals. Frequencies of T-bet+ tetramer+ events are shown. B) The frequencies of T-bet expression among tetramer+ SIV-specific CD8+ T cells in mLN, sLN, blood and spleen at the indicated time points; Linear regression ANOVA for samples collected between 13dpi and 90dpi. Lines connecting data points represent longitudinally collected data from sLN and blood of individual animals. No significant differences “ns”.

To define potential mechanisms underlying the loss of cytolytic molecule expression, we assessed T-bet expression in tetramer+ SIV-specific CD8+ T cells from acute through chronic infection. T-bet was initially expressed by a subset of SIV-specific CD8+ T cells in blood, LN, and spleen (Fig 3a) and gut mucosa (S4a Fig) during acute infection. T-bet expression in SIV-specific CD8+ T cells peaked at 13dpi in all tissues except the spleen, where peak T-bet expression was more varied (Fig 3b and S4b Fig). Subsequent to peak expression, T-bet expression in TL8-specific CD8+ T cells in LN and blood declined precipitously following peak viremia, in a similar fashion as cytolytic molecules in SIV-specific CD8+ T cells declined (Fig 2c). Similar, though not as dramatic, losses of T-bet were also observed in CM9-specific CD8+ T cells following peak viremia.

We further examined T-bet expression within SIV-specific CD8+ T cells that co-expressed perforin and granzyme B. T-bet expression was higher in Perf+GrzB+ SIV-specific CD8+ T cells in all sites during the acute period except in spleen, where T-bet expression varied (S6a Fig). Following the acute period, T-bet expression within Perf+GrzB+ SIV-specific CD8+ T cells declined as infection progressed, with the strongest effect occurring in TL8-specific responses. At 90dpi, similarly low levels of T-bet were detected between the different antigen specificities and tissues examined. Together these data show that SIV-specific CD8+ T cells rapidly lose expression of the master transcription factor T-bet that is likely required to sustain optimal cytolytic responses within CD8+ T cells during the course of infection.

### Partial loss of the cytolytic profile in responding SIV-specific CD8+ T cells following acute SIV infection

We next assessed how SIV-specific CD8+ T cells capable of responding to stimulation with SIV peptides changed over time, and whether these cells expressed granzyme B and T-bet. SIV-specific CD8+ T cell functional responses to Tat-TL8 or Gag-CM9 peptides peaked between 13 and 20dpi in SLT and blood of most animals as shown in S7a Fig, similar to the kinetics of Mamu A*01 tetramer binding (Fig 2a), then subsequently declined in most animals as infection progressed. One animal maintained elevated frequencies of CM9- specific functional CD8+ T cell responses up until necropsy at 90dpi.

**Fig 4:**
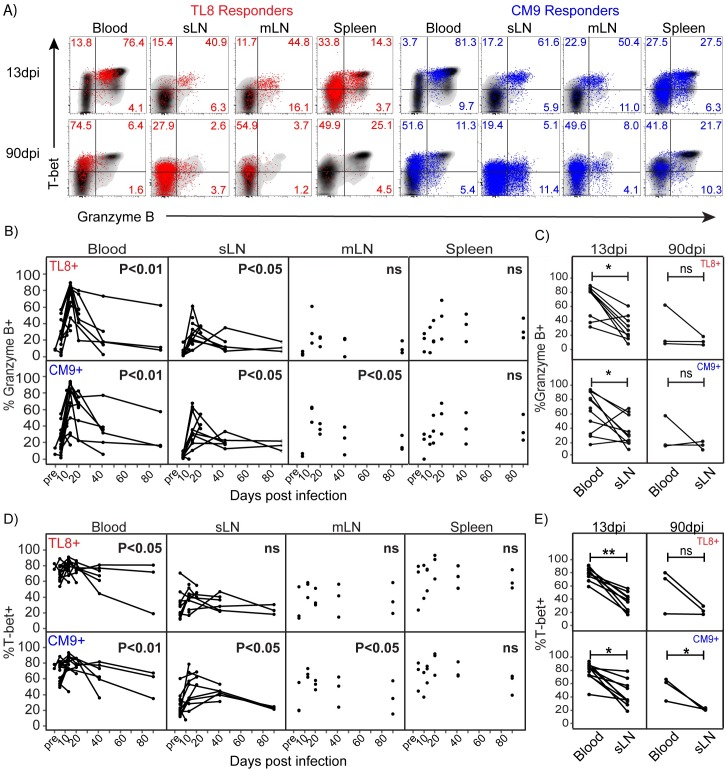
Functional SIV-specific CD8+ T cell responses lose cytolytic profiles throughout acute infection. A) Flow cytometry plots of granzyme B and T-bet expression in TL8 and CM9 functional (CD107a+ and/or IFNg+ and/or TNF+) SIV-specific CD8+ T cells (TL8 responder in red, and CM9 responder in blue) overlayed on total CD8+ T cells (black) in blood, sLN, mLN and spleen at the indicated time points from representative animals. B and D) Kinetics of granzyme B (B) and T-bet (D) expression in functional TL8 and CM9-specific CD8+ T cell responses in mLN, sLN, blood and spleen at the indicated time points. Linear regression ANOVA for samples collected between 13dpi and 90dpi. C and E) Frequencies of granzyme B (C) and T-bet (E) expression in functional TL8 and CM9-specific CD8+ T cell responses in matched sLN and blood samples at 13 or 90dpi. Paired, one-tailed Student’s t-test; 13dpi (TL8; n = 9 pairs and CM9; n = 12 pairs) and 90dpi (TL8 and CM9; n = 3 pairs). Lines connecting data points represent longitudinally collected data from sLN and blood of individual animals. * p≤0.05, ** p<0.01. No significant differences “ns”.

Granzyme B was highly expressed in functional SIV-specific CD8+ T cells peaking between 13 and 20dpi in LN, spleen and blood, as shown in Fig 4a and 4b, similar to granzyme B expression kinetics within Perf+GrzB+ tetramer+ SIV-specific CD8+ T cells (Fig 2c). Detection of perforin expression was not performed in CD8+ T cells following SIV peptide stimulation due to lack of appropriate reagents to detect newly upregulated perforin following stimulation. Granzyme B expression in responding SIV-specific CD8+ T cells was 2-fold higher in blood compared to matched sLN during acute infection (Fig 4c). Collectively, these data indicate that responding subsets of SIV-specific CD8+ T cells display rapid loss of cytolytic protein expression similar to the total SIV-specific CD8+ T cell population as infection progresses.

### Tissue-specific T-bet dysregulation is associated with declining cytolytic SIV-specific CD8+ T cell functional responses during acute infection

We next evaluated T-bet expression in functional SIV-specific CD8+ T cell responses in blood, sLN, mLN, and spleen as shown in Fig 4a. We observed similar T-bet expression kinetics among the functional subset of SIV-specific CD8+ T cells as compared to the total tetramer+ responses in LN, where declining expression followed peak acute phase at 13dpi (Figs 3b and 4d). However, unlike total tetramer+ SIV-specific CD8+ T cells from blood, the functional subset of SIV-specific CD8+ T cells from blood expressed higher levels of T-bet at peak response, which declined somewhat as infection progressed during the contraction of functional responses to chronic SIV infection (Figs 3b and 4d). Unlike total tetramer+ SIV-specific CD8+ T cells, T-bet expression was significantly higher in blood as compared to sLN during acute infection, and in CM9-specific responses during chronic infection (Fig 4e). T-bet expression was also higher in TL8-specific responses from blood during chronic infection in 2 out of 3 animals. The loss of T-bet in functional cells over time also extended to granzyme B expressing subsets within the SIV-specific CD8+ T cells in nearly all animals in all tissues (S7b Fig). These data show that the association between T-bet and granzyme B expression in the functional subsets SIV-specific CD8+ T cells is heightened in circulating responses compared to LN.

We next evaluated the memory subset distribution of SIV-specific CD8+ T cells in blood, sLN, mLN, spleen and rectal biopsies throughout acute infection (S8a and S8b Fig). SIV-specific CD8+ T cells in peripheral blood and spleen during the peak response were predominately effector memory phenotype. As infection progressed, the frequencies of effector memory SIV-specific CD8+ T cells in peripheral blood and spleen declined. Low levels of effector memory phenotype SIV-specific CD8+ T cells were found in sLN, mLN, and gut mucosa among SIV-specific CD8+ T cells throughout infection. These data indicate low levels of effector memory SIV-specific CD8+ T cells in LN tissues throughout the acute phase of infection in close proximity to cells harboring virus.

### Transcriptional requirement for maintenance of granzyme B expression following degranulation is dysregulated during chronic infection

**Fig 5:**
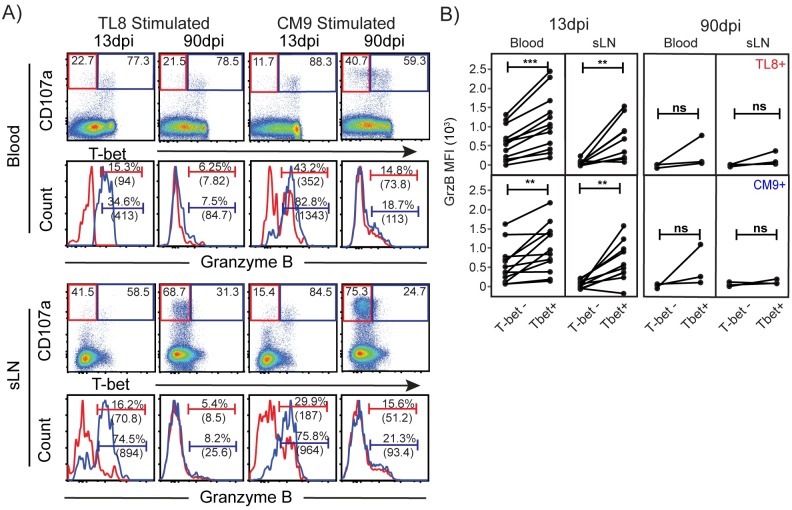
T-bet expression sustains cytolytic potential following degranulation. A) Flow cytometry plots of T-bet and CD107a staining in CD8+ T cells following *in vitro* SIV peptide (Tat-TL8 or Gag-CM9) stimulation at the indicated time points from blood and sLN. Gated frequencies represent the percentage of T-bet+ or T-bet-events among CD107a bright events. Histograms of granzyme B expression in CD107a bright, T-bet+ (blue) and T-bet- (red) CD8+ T cell responses corresponding to the above flow cytometry plot. Gated regions indicate the frequency of granzyme B+ events (MFI of granzyme B in parenthesis) from the indicated parent gate above. B) MFI of granzyme B in matched CD107a bright, T-bet+ and T-bet-, events from blood and sLN at the indicated time points. Paired, one-tailed Student’s t-test; 13dpi (n = 9 to 12 pairs) and 90dpi (n = 3pairs). * p≤0.05, ** p<0.01, and ***p<0.0001. No significant differences “ns”.

To evaluate the role of T-bet in the maintenance of granzyme B within functional SIV-specific CD8+ T cells, we assessed whether granzyme B expression in degranulating SIV-specific CD8+ T cells was preferentially maintained in T-bet expressing cells. As shown in Fig 5a and 5b, degranulating SIV-specific CD8+ T cells that express T-bet were significantly higher in granzyme B expression levels during the acute phase in both blood and sLN compared to T-bet negative cells. However, after establishment of chronic infection, this relationship was lost. Regardless of T-bet expression, degranulating SIV-specific CD8+ T cells fail to express high levels of granzyme B in both blood and sLN. Similar observations were made for both TL8 and CM9-specific CD8 responses indicating that this loss of proper regulation of cytolytic factor expression occurs independent of epitope specificity and viral escape patterns.

## Discussion

It has long been appreciated that CD8+ T cells play a critical role in controlling viremia during acute and chronic SIV infection. However, the mechanisms underlying the ineffectiveness of CD8+ T cells to mediate control as disease progresses have remained unclear. Recent studies of acute HIV infection from our group have highlighted cytolytic HIV/SIV-specific CD8+ T cell responses developing in peripheral blood very early in acute infection, but subsequently losing cytolytic factor expression following resolution of acute viremia [18, 38]. To date, most studies have primarily characterized CD8+ T cell responses from peripheral blood; however, viral replication occurs primarily in tissues such as SLT and gut mucosa. As such, it is unknown if HIV/SIV-specific cytotoxic CD8+ T cell responses are present in critical anatomical locations during acute infection, and if so, whether these responses decline or are maintained after acute infection.

In the current study, we investigated the evolution of SIV-specific CD8+ T cells from blood, LN, spleen and gut mucosa of SIV infected RM during acute infection and through the transition to the chronic phase. We found that the cytolytic proteins perforin and granzyme B were initially expressed in SIV-specific CD8+ T cells in tissues where viral replication and reservoir are established during the earliest phases of infection. However, this early expression of cytolytic proteins collapsed following peak CD8+ T cell response in all anatomical sites as infection progressed, due in part to an inability to maintain high levels of the transcription factor T-bet, a known regulatory factor of CD8+ T cell cytolytic functions. This collapse in T-bet driven cytolytic profile may additionally be influenced by expansion of alternate epitope specific responses and contraction of primary responses such as TL8 or CM9. Overall, the short-lived cytolytic profile of SIV-specific responses within tissues suggests that cytotoxic T lymphocyte dysregulation is initiated in the earliest responses with suboptimal induction of cytolytic factor expression in SIV-specific CD8+ T cells during acute infection leading to insufficient maintenance of CTL responses.

In addition to declining expression of cytolytic proteins in SIV-specific CD8+ T cells following peak viremia, we found tissue-specific disparities in expression of perforin, granzyme B, and the transcription factor T-bet that provide some resolution to the previously described observation of poor effector functions in CD8+ T cells from lymph node tissue during chronic HIV infection [36]. Our detection of both lower T-bet and cytolytic protein expression in the earliest SIV-specific CD8+ T cells in sLN as compared to blood suggest tissue-specific downregulation of cytolytic factor expression and/or impaired replenishment following degranulation in these responses. Importantly, these tissue-specific expression profiles are detected in both TL8 and CM9-specific CD8+ T cell responses, possibly supportive of extrinsic, tissue-induced regulation of T-bet as opposed to intrinsic regulation mediated by TCR signaling or availability of cognate antigen. These results suggest that SLTs provide an environment where CD8+ T cell ability to directly eliminate infected cells is hampered, favoring establishment and persistence of HIV reservoirs. As such, it is likely that CD8+ T cell surveillance within lymphoid tissues is mediated in part by non-cytolytic CD8+ T cell functions [11, 12].

Declining levels of T-bet in peripheral blood virus-specific CD8+ T cells have been shown to parallel the loss of cytolytic factor expression and disease progression in chronic HIV, HCV and HBV infections [18, 48]. The early induction of perforin and granzyme B co-expression in SIV-specific CD8+ T cells in the absence of high levels of T-bet and even in T-bet deficient cells, suggest that alternate mechanisms are likely involved in regulating the initial cytolytic program. This alternatively regulated induction may be driven by other transcription factors including Eomes, Blimp1, or Runx3 as previously described [17, 49]. Importantly, the short-lived co-expression of perforin and granzyme B implies that initial high and persistent levels of T-bet, may be necessary to maintain cytolytic factor expression. Indeed, while SIV-specific CD8+ T cell responses expressing T-bet initially maintained granzyme B expression following degranulation in responses from SLT and blood, later in infection T-bet expression became de-coupled from granzyme B re-expression in stimulated SIV-specific CD8+ T cells. These results point to additional regulatory deficiencies within SIV-specific CD8+ T cells that result in an inability to maintain cytolytic activity.

It is well accepted that T cell exhaustion occurs in chronic HIV/SIV infection [50–53]. However, it is unclear how early manifestations of exhaustion first appear. Experiments in mouse models suggest that even during the acute phase of clone13 chronic LCMV infection, there are early indicators of exhaustion [54]. Here, by 90 dpi, we can already observe an inability of SIV-specific CD8+ T cells to upregulate granzyme B after stimulation. A similar observation was previously made in chronic HIV infection by Migueles and colleagues [25]. Importantly, we now show that this develops extremely rapidly, in both blood and lymphoid tissue, and that at least one cause of this dysfunction is the de-coupling of granzyme B expression from T-bet regulation. While we did not measure markers of exhaustion, such as PD-1, due to their association with T cell activation during acute infection [54–56] it is clear that this de-coupling is not simply due to chronic antigen-specific stimulation, as the same inabilities are manifest in the Tat-TL8 response, which escapes by 20dpi, and the Gag-CM9 response which does not escape. Importantly, having now found that this functional disparity also occurs in the RM SIV infection model, we can begin to explore interventional means to prevent or reverse this dysfunction and thereby determine whether maintaining properly regulated cytolytic effector functions in SIV-specific CD8+ T cells can modulate progression outcome.

Here, we have described for the first time the very early, uniform, and precipitous loss of granule-mediated cytolytic properties of SIV-specific CD8+ T cells that occurs not only in peripheral blood, but also at key viral replication and reservoir sites within lymphoid tissue and gut mucosa. The inability for SLT and gut mucosa SIV-specific CD8+ T cells to highly express and effectively maintain cytolytic properties may foster seeding of viral reservoir and replication in lymphoid follicles under limited immune pressures. These results have broad implication in the setting of therapeutic HIV reservoir reactivation strategies, as even re-invigorated HIV/SIV-specific CD8+ T cell responses in lymphoid tissue may inherently not be capable of eliminating reactivated latently infected CD4+ T cells. More generally, these findings may address important features of CD8+ T cell differentiation that limit full acquisition and maintenance of effector capacity within critical sites for HIV-specific immune surveillance.

## Supporting Information

S1 Fig Established infection and sample collection schedule. A) Detailed tissue collection schedule including the number of unique samples collected at each indicated time point. B) SIV blood plasma viral loads (RNA copies/mL) at the indicated time points post-infection from each animal. Lines connecting data points reflect longitudinal monitoring of individual animals. C) Absolute CD4+ T cell counts per mL of whole blood at the indicated time points from individual animals. Mean ± SD of data collected at each time point is plotted as a line along with individual data points. D) CD4+ T cell counts as a proportion of total CD3+ T lymphocytes from rectal biopsies (RB) at the indicated time points from individual animals. Mean ± SD of data collected at each time point is plotted as a line along with individual data points.
(PDF)

S2 Fig Sequence evolution of Tat-TL8 and Gag-CM9 epitopes. A) Amino acid sequences of the six most frequently represented Tat-TL8 and Gag-CM9 epitope sequence variants within SIV_mac251_ stock inoculum virus. Canonical Tat-TL8 eptiope sequence is highlighted in red, and Gag-CM9 epitope sequence in blue. Canonical Tat-SL8 SIVmac239 epitope sequence is highlighted in pink. B) Amino acid sequences of the six most frequently represented Tat-TL8 and Gag-CM9 epitope sequence variants detected from blood plasma of animals RGl14 and RHk14 at the indicated time points post infection. Frequencies of reads corresponding to the indicated epitope variant are indicated. C) Frequencies of canonical TL8 and CM9 epitope sequences in plasma virus throughout infection until necropsy at 90dpi from two representative animals. Dashed line indicates the frequency of canonical TL8 or CM9 epitope detected in inoculum viral stock as shown in A.
(PDF)

S3 Fig SIV-specific responses are detected in SLT and gut mucosa by Mamu A*01 tetramer staining. A) Flow cytometry plots of Mamu A*01 Tat-TL8 and Gag-CM9 tetramer staining in sLN, mLN, spleen and RB at the indicated time points from representative animals. Frequencies of gated events are shown.
(PDF)

S4 Fig Granzyme B and T-bet expression are detectable in SIV-specific CD8+ T cells from gut mucosa during acute SIV infection. A) Flow cytometry plots of granzyme B and T-bet expression in TL8 and CM9-specific CD8+ T cells (Tat-TL8 tetramer+ CD8+ T cells in red and Gag-CM9 tetramer+ responses blue) in total CD8+ T cells (black) from RB samples pre-infection, at 13dpi, and 41dpi (late acute) from a representative animal. Frequencies of gated events among tetramer+ events are shown. B) Kinetics of granzyme B and T-bet expression in tetramer+ SIV-specific CD8+ T cells from RB throughout acute infection. Lines connecting data points represent longitudinally collected data from individual animals.
(PDF)

S5 Fig Kinetics of granzyme B MFI in cytolytic tetramer+ SIV-specific CD8+ T cells during acute infection. Median fluorescence intensity (MFI) of granzyme B in Perf+GrzB+ TL8 and CM9 tetramer+ SIV-specific CD8+ T cells in blood, sLN, mLN, and spleen at the indicated throughout infection. Linear regression ANOVA for samples collected between 13dpi and 90dpi. Lines connecting data points represent longitudinally collected data from sLN and blood of individual animals. No significant differences “ns”.
(PDF)

S6 Fig Kinetics of T-bet expression in tetramer+ SIV-specific CD8+ T cells throughout acute infection. A) Frequencies of T-bet expression in cytolytic (Perf+GrzB+) SIV-specific CD8+ T cells in blood, sLN, mLN and spleen throughout infection. Linear regression ANOVA for samples collected between 13dpi and 90dpi. Lines connecting data points represent longitudinally collected data from sLN and blood of individual animals. No significant differences “ns”.
(PDF)

S7 Fig Functional SIV-specific CD8+ T cell responses throughout infection. A) Frequencies of Tat-TL8 and Gag-CM9 functional CD8+ T cell responses in total CD8+ T cells following *in vitro* stimulation from blood, sLN, mLN and spleen at the indicated time points. B) Frequencies of T-bet expression in cytolytic (GrzB+) functional SIV-specific CD8+ T cell responses from blood, sLN, mLN and spleen at the indicated time points. Linear regression ANOVA for samples collected between 13dpi and 90dpi. C) Frequencies of T-bet expression in cytolytic SIV-specific CD8+ T cell responses in matched sLN and blood at 13dpi and 90dpi. Paired, one-tailed Student’s t-test; 13dpi (n = 10 to 12 pairs) and 90dpi (n = 3pairs). Lines connecting data points represent longitudinally collected data from sLN and blood of individual animals. * p≤0.05, ** p<0.01. No significant differences “ns”.
(PDF)

S8 Fig Memory distribution of SIV-specific CD8+ T cells throughout acute infection. A) Flow cytometry plots of CD28 and CD95 expression in TL8 and CM9-specific CD8+ T cells (Tat-TL8 tetramer+ CD8+ T cells in red and Gag-CM9 tetramer+ responses blue) in total CD8+ T cells (black) from all tissues at the indicated time points pre- and post-infection. Frequencies of gated events among tetramer+ events are shown. Right: legend for memory subsetting of CD8+ T cells by CD28, CD95 and CCR7. B) Frequencies of central memory (black dashed line), transitional memory (green dotted line) and effector memory (solid blue line) differentiated SIV-specific CD8+ T cells in the indicated tissues throughout infection. Lines represent mean for the collected samples at the indicated time points.
(PDF)
