# Supplementary material for: Collapse of Cytolytic Potential in SIV-Specific CD8+ T Cells Following Acute SIV Infection in Rhesus Macaques
Source: PLoS Pathog. 2016 Dec 30;12(12):e1006135. doi: 10.1371/journal.ppat.1006135 (PMC5231392; doi:10.1371/journal.ppat.1006135)

Supplemental Figure 1

A)

Tissue Collected (TC)	Tissue collection and necropsy timepoints Days post infection							
	-36	0	5	10	13	20	41	90
Lymph Node	18	-	18	6	12	3	6	3
Blood	18	-	18	15	12	9	6	3
Rectal Biopsy	18	-	15	-	9	-	3	-
CBC/Viral load	18	-	18	15	12	9	6	3
Necropsy (Nec)	-	-	3	3	3	3	3	3

B)

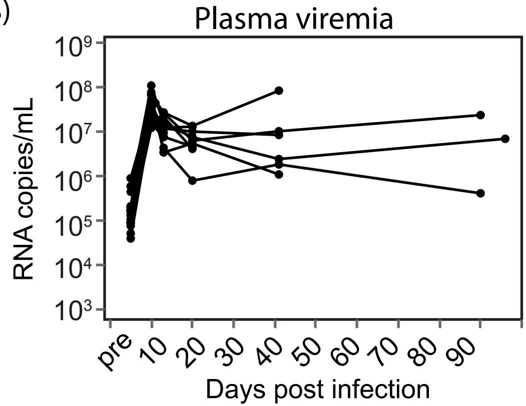

C)

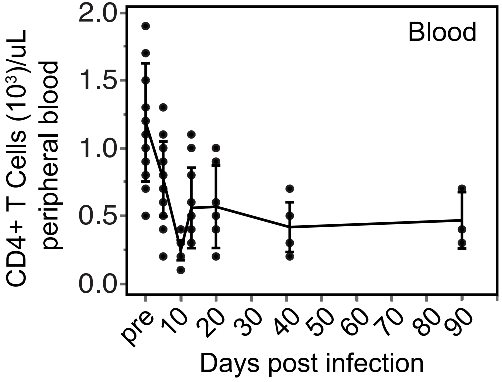

D)

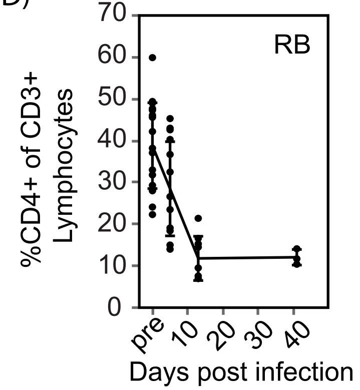

Supplement: S1 Fig — A) Detailed tissue collection schedule including the number of unique samples collected at each indicated time point. B) SIV blood plasma viral loads (RNA copies/mL) at the indicated time points post-infection from each animal. Lines connecting data points reflect longitudinal monitoring of individual animals. C) Absolute CD4+ T cell counts per mL of whole blood at the indicated time points from individual animals. Mean ± SD of data collected at each time point is plotted as a line along with individual data points. D) CD4+ T cell counts as a proportion of total CD3+ T lymphocytes from rectal biopsies (RB) at the indicated time points from individual animals. Mean ± SD of data collected at each time point is plotted as a line along with individual data points. (PDF) [file ppat.1006135.s001.pdf]
