# Supplementary material for: Collapse of Cytolytic Potential in SIV-Specific CD8+ T Cells Following Acute SIV Infection in Rhesus Macaques
Source: PLoS Pathog. 2016 Dec 30;12(12):e1006135. doi: 10.1371/journal.ppat.1006135 (PMC5231392; doi:10.1371/journal.ppat.1006135)
Supplement: S2 Fig — A) Amino acid sequences of the six most frequently represented Tat-TL8 and Gag-CM9 epitope sequence variants within SIVmac251 stock inoculum virus. Canonical Tat-TL8 eptiope sequence is highlighted in red, and Gag-CM9 epitope sequence in blue. Canonical Tat-SL8 SIVmac239 epitope sequence is highlighted in pink. B) Amino acid sequences of the six most frequently represented Tat-TL8 and Gag-CM9 epitope sequence variants detected from blood plasma of animals RGl14 and RHk14 at the indicated time points post infection. Frequencies of reads corresponding to the indicated epitope variant are indicated. C) Frequencies of canonical TL8 and CM9 epitope sequences in plasma virus throughout infection until necropsy at 90dpi from two representative animals. Dashed line indicates the frequency of canonical TL8 or CM9 epitope detected in inoculum viral stock as shown in A. (PDF) [file ppat.1006135.s002.pdf]

Supplemental Figure 2

A) SIVmac251 Inoculum

Tat-TL8	TTPESANL	53.8%
	STPESANL	36.2%
	NTPESANL	3.1%
	PTPESANL	3.0%
	STPELANL	1.6%
	ATPESANL	1.2%
Gag-CM9	CTPYDINQM	97.4%
	YTPYDINQM	1.0%
	CPPYDINQM	0.3%
	CAPYGINQM	0.3%
	CTHYDINQM	0.3%
	CTPYDNNQI	0.3%

C)

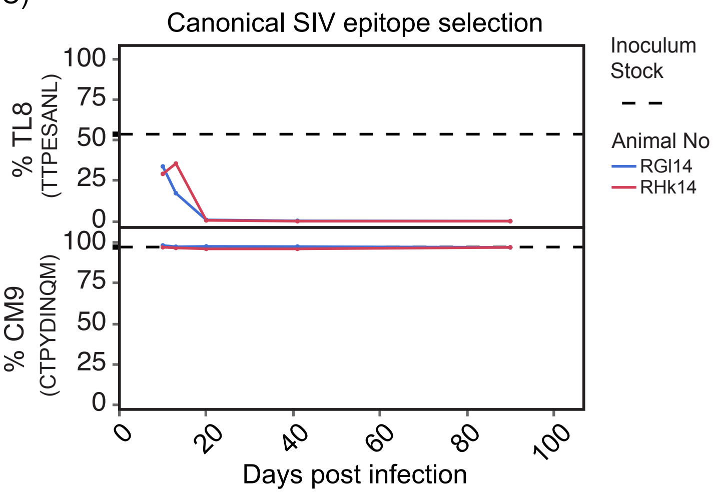

B)

Tat-TL8 (WT-TTPESANL)

Animal No.	10dpi		13dpi		20dpi		41dpi		90dpi	
RGI14	STPESANL	52.4%	STPESANL	49.0%	PTPESANL	46.7%	NTPESANL	60.4%	NTPESANL	43.2%
	TTPESANL	33.8%	PTPESANL	28.9%	NTPESANL	28.8%	PTPESANL	23.94%	PTPESANL	38.4%
	PTPESANL	11.4%	TTPESANL	17.3%	STPESANL	17.7%	STPESANL	12.5%	STPESANL	17.2%
	NTPESANL	1.2%	NTPESANL	3.1%	SIPESANL	1.8%	TTSESANL	1.1%	STPESANR	0.1%
	ATPESANL	0.2%	TTPESTNL	0.2%	SAPESANL	0.9%	TTPESANL	0.2%	STPESTNL	0.1%
	STPESVNL	0.1%	ATPESANL	0.1%	TTPESANL	0.8%	SIPESANL	0.2%	PTPESANL	0.1%
RHk14	STPESANL	57.7%	STPESANL	49.5%	PTPESANL	42.2%	PTPESANL	43.0%	STPELANL	39.2%
	TTPESANL	29.2%	TTPESANL	35.6%	STPESANL	27.9%	STPELANL	33.1%	PTPESANL	31.6%
	PTPESANL	6.5%	PTPESANL	6.5%	STPELANL	21.2%	STPESANL	15.2%	STPESANL	11.9%
	STPELANL	2.2%	STPELANL	2.2%	NTPESANL	2.8%	PTPELANL	2.4%	SIPESANL	7.6%
	NTPESANL	1.0%	NTPESANL	1.0%	PTPELANL	0.6%	TIPESANL	0.6%	PTPELANL	5.1%
	ATPESANL	0.4%	STLESANL	0.4%	TTPESANL	0.5%	STPESANP	0.6%	PTPESTNL	1.1%

Gag-CM9 (WT-CTPYDINQM)

Animal No.	10dpi		13dpi		20dpi		41dpi		90dpi	
RGI14	CTPYDINQM	98.1%	CTPYDINQM	97.3%	CTPYDINQM	97.5%	CTPYDINQM	97.4%	CTPYDINQM	96.9%
	CIPYDINQM	0.2%	YTPYDINQM	0.3%	CAPYDINQM	0.3%	RTPYDINQM	0.3%	YTPYDINQM	0.4%
	YTPYDINQM	0.2%	RTPYDINQM	0.2%	RTPYDINQM	0.2%	YTPYDINQM	0.2%	CTHYDINQM	0.2%
	CTSYDINQM	0.1%	CTLYDINQM	0.2%	YTPYDINQM	0.2%	CTPYDINQI	0.2%	RTPYDINQM	0.2%
	RTPYDINQM	0.1%	CSPYDINQM	0.2%	CTHYDINQM	0.2%	CTPYDINQV	0.1%	CPPYDINQM	0.1%
	CTPYDIDQM	0.1%	CTPYDIN*M	0.2%	CTSYDINQM	0.2%	CIPYDINQM	0.1%	CTAYDINQM	0.1%
RHk14	CTPYDINQM	97.0%	CTPYDINQM	96.6%	CTPYDINQM	96.0%	CTPYDINQM	96.0%	CTPYDINQM	96.9%
	RTPYDINQM	0.5%	CTPYDVNQM	0.4%	CAPYDINQM	0.6%	CTPYDINQQ	0.7%	CAPYDINQM	0.4%
	YTPYDINQM	0.4%	CAPYDINQM	0.3%	YTPYDINQM	0.5%	YTPYDINQM	0.5%	RTPYDINQM	0.3%
	CTTYDINQM	0.4%	RTPYDINQM	0.2%	CTPHDINQM	0.3%	RTPYDINQM	0.3%	YTPYDINQM	0.2%
	CSPYDINQM	0.3%	CTP*DINQM	0.2%	CTPYGINQM	0.3%	CAPYDINQM	0.3%	CSPYDINQM	0.2%
	CAPYDINQM	0.2%	CTPYGINQM	0.2%	*TPYDINQM	0.1%	CTPYGINQM	0.3%	CPPYDINQM	0.2%
