# Supplementary figures and images for: Collapse of Cytolytic Potential in SIV-Specific CD8+ T Cells Following Acute SIV Infection in Rhesus Macaques
Source: PLoS Pathog. 2016 Dec 30;12(12):e1006135. doi: 10.1371/journal.ppat.1006135 (PMC5231392; doi:10.1371/journal.ppat.1006135)

# Supplemental Figure 3

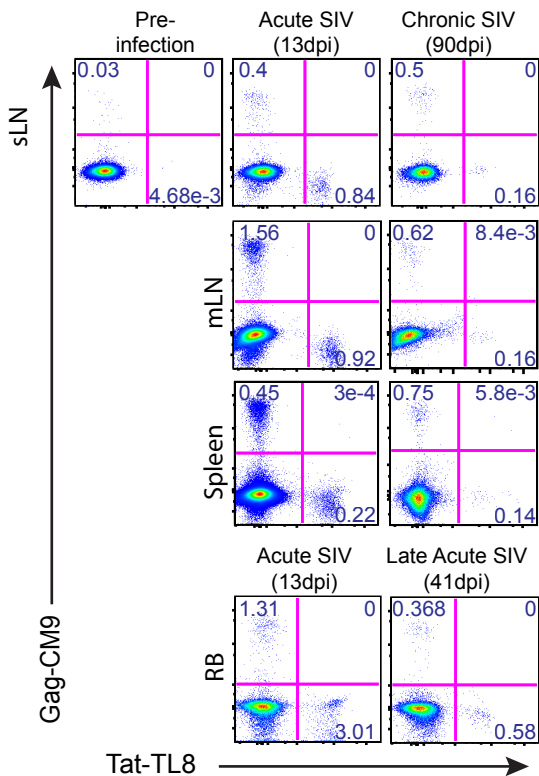

Supplement: S3 Fig — A) Flow cytometry plots of Mamu A*01 Tat-TL8 and Gag-CM9 tetramer staining in sLN, mLN, spleen and RB at the indicated time points from representative animals. Frequencies of gated events are shown. (PDF) [file ppat.1006135.s003.pdf]

# Supplemental Figure 4

A)

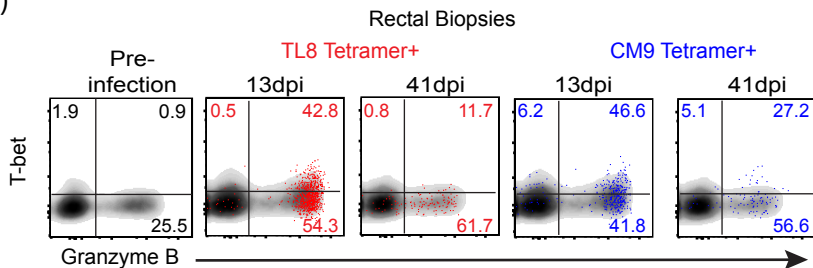

B)

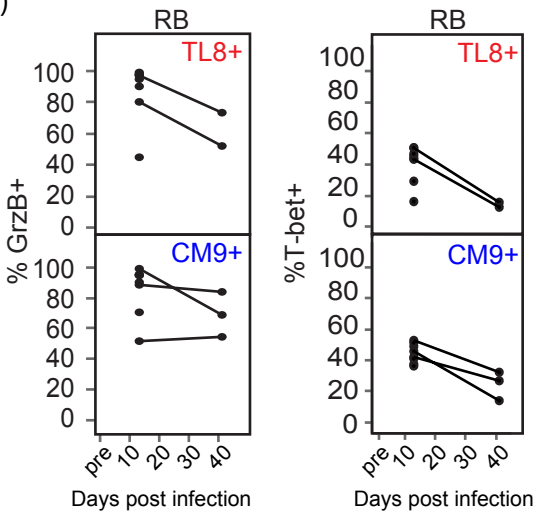

Supplement: S4 Fig — A) Flow cytometry plots of granzyme B and T-bet expression in TL8 and CM9-specific CD8+ T cells (Tat-TL8 tetramer+ CD8+ T cells in red and Gag-CM9 tetramer+ responses blue) in total CD8+ T cells (black) from RB samples pre-infection, at 13dpi, and 41dpi (late acute) from a representative animal. Frequencies of gated events among tetramer+ events are shown. B) Kinetics of granzyme B and T-bet expression in tetramer+ SIV-specific CD8+ T cells from RB throughout acute infection. Lines connecting data points represent longitudinally collected data from individual animals. (PDF) [file ppat.1006135.s004.pdf]

Supplemental Figure 5

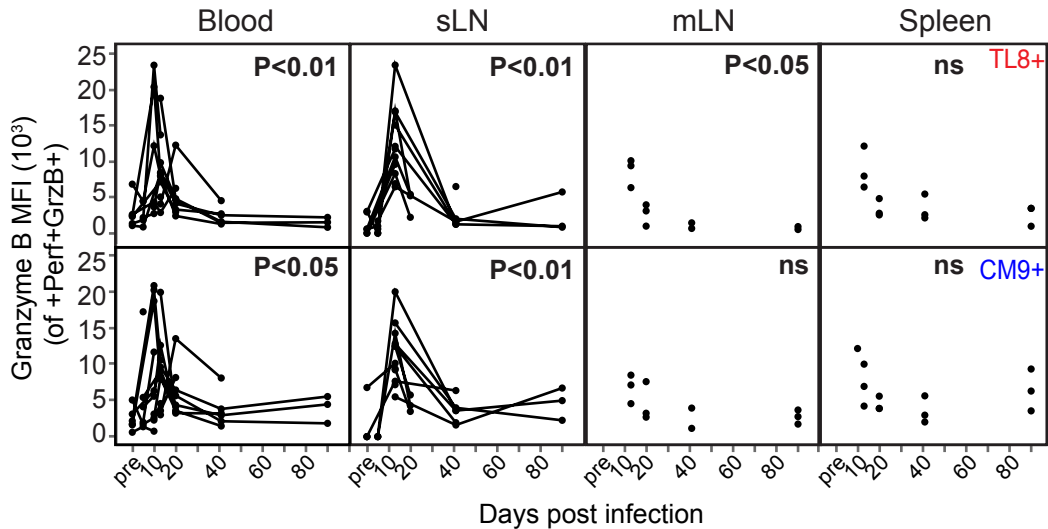

Supplement: S5 Fig — Median fluorescence intensity (MFI) of granzyme B in Perf+GrzB+ TL8 and CM9 tetramer+ SIV-specific CD8+ T cells in blood, sLN, mLN, and spleen at the indicated throughout infection. Linear regression ANOVA for samples collected between 13dpi and 90dpi. Lines connecting data points represent longitudinally collected data from sLN and blood of individual animals. No significant differences “ns”. (PDF) [file ppat.1006135.s005.pdf]

## Supplemental Figures 6

A)

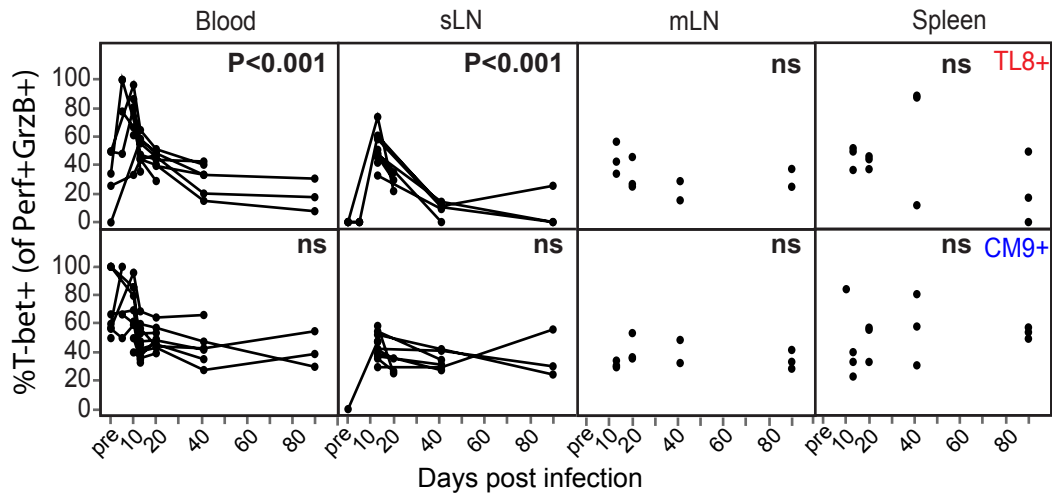

Supplement: S6 Fig — A) Frequencies of T-bet expression in cytolytic (Perf+GrzB+) SIV-specific CD8+ T cells in blood, sLN, mLN and spleen throughout infection. Linear regression ANOVA for samples collected between 13dpi and 90dpi. Lines connecting data points represent longitudinally collected data from sLN and blood of individual animals. No significant differences “ns”. (PDF) [file ppat.1006135.s006.pdf]

Supplemental Figure 7

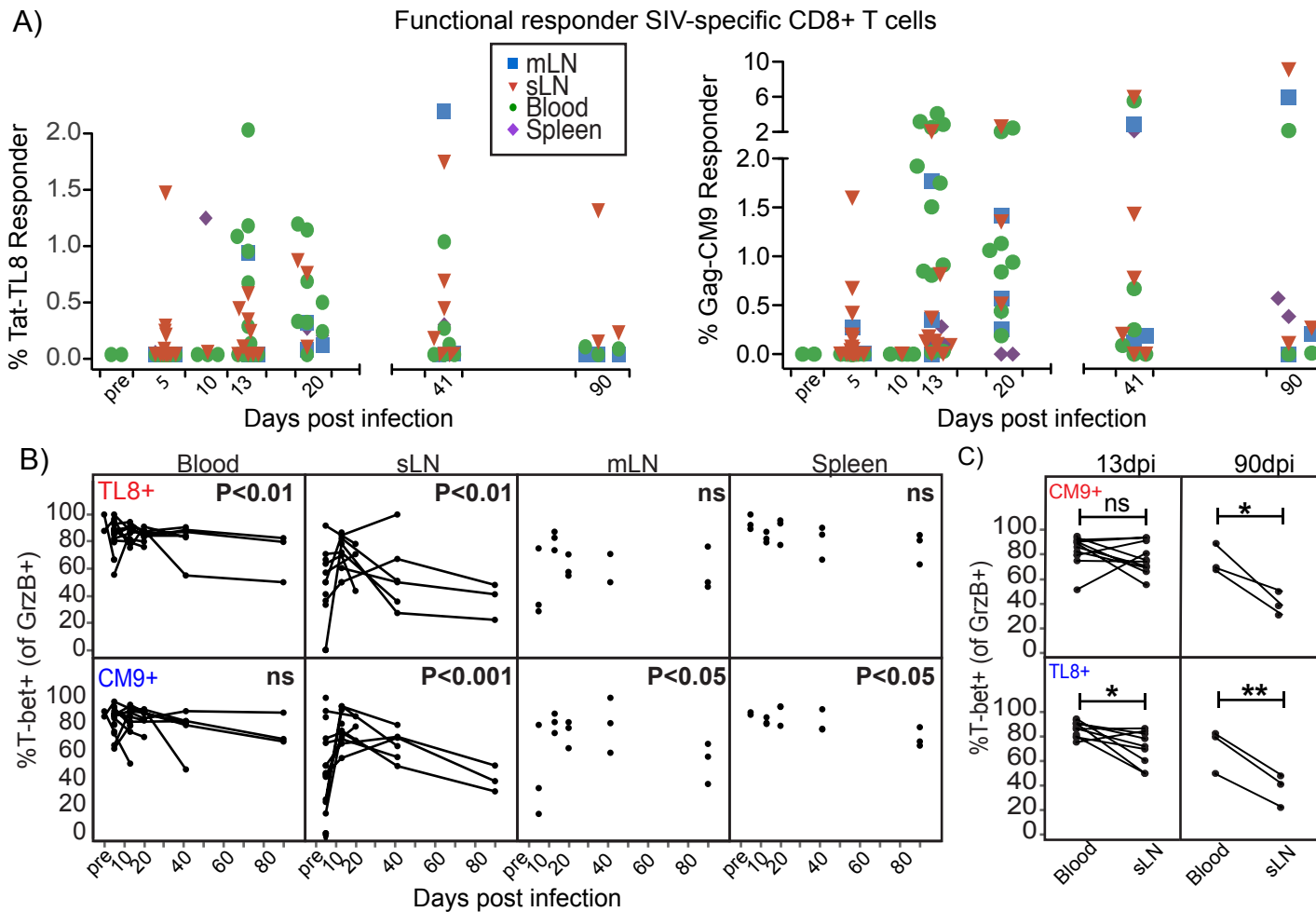

Supplement: S7 Fig — A) Frequencies of Tat-TL8 and Gag-CM9 functional CD8+ T cell responses in total CD8+ T cells following in vitro stimulation from blood, sLN, mLN and spleen at the indicated time points. B) Frequencies of T-bet expression in cytolytic (GrzB+) functional SIV-specific CD8+ T cell responses from blood, sLN, mLN and spleen at the indicated time points. Linear regression ANOVA for samples collected between 13dpi and 90dpi. C) Frequencies of T-bet expression in cytolytic SIV-specific CD8+ T cell responses in matched sLN and blood at 13dpi and 90dpi. Paired, one-tailed Student’s t-test; 13dpi (n = 10 to 12 pairs) and 90dpi (n = 3pairs). Lines connecting data points represent longitudinally collected data from sLN and blood of individual animals. * p≤0.05, ** p<0.01. No significant differences “ns”. (PDF) [file ppat.1006135.s007.pdf]

# Supplemental Figure 8

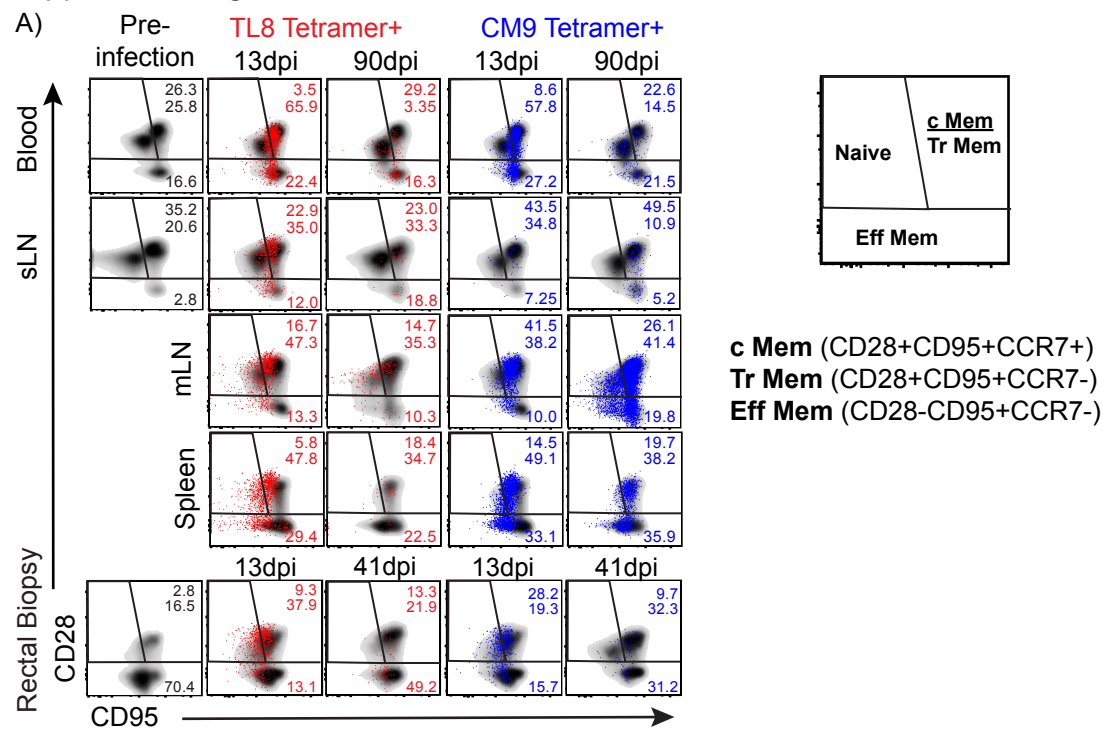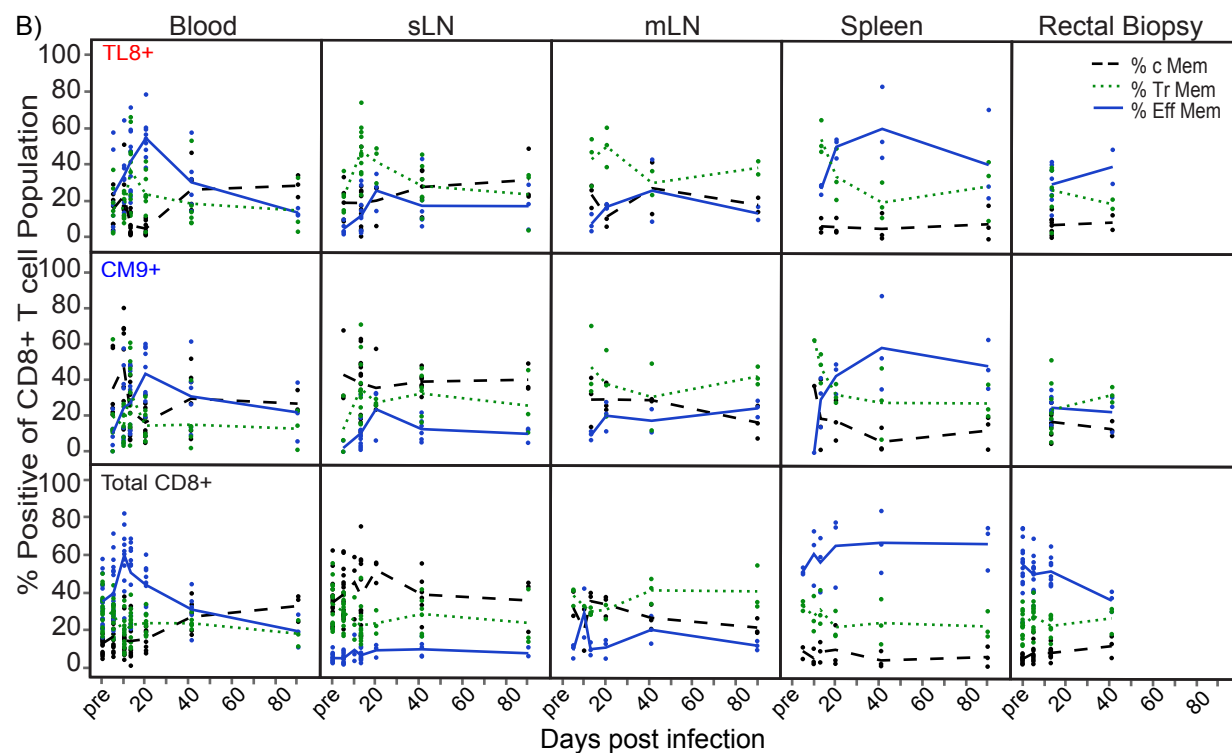

Supplement: S8 Fig — A) Flow cytometry plots of CD28 and CD95 expression in TL8 and CM9-specific CD8+ T cells (Tat-TL8 tetramer+ CD8+ T cells in red and Gag-CM9 tetramer+ responses blue) in total CD8+ T cells (black) from all tissues at the indicated time points pre- and post-infection. Frequencies of gated events among tetramer+ events are shown. Right: legend for memory subsetting of CD8+ T cells by CD28, CD95 and CCR7. B) Frequencies of central memory (black dashed line), transitional memory (green dotted line) and effector memory (solid blue line) differentiated SIV-specific CD8+ T cells in the indicated tissues throughout infection. Lines represent mean for the collected samples at the indicated time points. (PDF) [file ppat.1006135.s008.pdf]
